# Ebola virus inclusion bodies are liquid organelles whose formation is facilitated by nucleoprotein oligomerization

**DOI:** 10.1080/22221751.2023.2223727

**Published:** 2023-06-22

**Authors:** Bianca S. Bodmer, Melina Vallbracht, Dmitry S. Ushakov, Lisa Wendt, Petr Chlanda, Thomas Hoenen

**Affiliations:** a Institute for Molecular Virology and Cell Biology, Friedrich-Loeffler-Institut, Greifswald - Insel Riems, Germany; b Schaller Research Groups, Department of Infectious Diseases, Virology, Heidelberg University Hospital, Heidelberg, Germany

**Keywords:** Ebolavirus, inclusion bodies, intrinsically disordered region, liquid organelle, liquid–liquid phase separation

## Abstract

Viral RNA synthesis of several non-segmented, negative-sense RNA viruses (NNSVs) takes place in inclusion bodies (IBs) that show properties of liquid organelles, which are formed by liquid–liquid phase separation of scaffold proteins. It is believed that this is driven by intrinsically disordered regions (IDRs) and/or multiple copies of interaction domains, which for NNSVs are usually located in their nucleo – and phosphoproteins. In contrast to other NNSVs, the Ebola virus (EBOV) nucleoprotein NP alone is sufficient to form IBs without the need for a phosphoprotein, and to facilitate the recruitment of other viral proteins into these structures. While it has been proposed that also EBOV IBs are liquid organelles, this has so far not been formally demonstrated. Here we used a combination of live cell microscopy, fluorescence recovery after photobleaching assays, and mutagenesis approaches together with reverse genetics-based generation of recombinant viruses to study the formation of EBOV IBs. Our results demonstrate that EBOV IBs are indeed liquid organelles, and that oligomerization but not IDRs of the EBOV nucleoprotein plays a key role in their formation. Additionally, VP35 (often considered the phosphoprotein-equivalent of EBOV) is not essential for IB formation, but alters their liquid behaviour. These findings define the molecular mechanism for the formation of EBOV IBs, which play a central role in the life cycle of this deadly virus.

## Introduction

Ebola virus (EBOV) is a non-segmented, negative-sense RNA virus (NNSV) that causes severe haemorrhagic fevers with high case fatality rates in humans. While EBOV-specific antibody-based therapeutics and vaccines have recently been approved, these are only directed against EBOV, but not against other highly pathogenic members of the ebolavirus genus [1, 2]. An approach directed at interfering with virus-host interactions or fundamental aspects of the EBOV life cycle that are common for all ebolaviruses could lead to the development of broadly active antivirals.

During infection with ebolaviruses, viral RNA synthesis (i.e. genome replication and transcription) takes place in inclusion bodies (IBs), which are distinct structures that form in the cytoplasm of infected cells [3, 4]. These structures are commonly formed during NNSV infection as sites of viral RNA synthesis [5–9]. Interestingly, while for other NNSVs both the nucleoprotein and phosphoprotein are the minimal components required for IB formation [5, 10, 11], for ebolaviruses the nucleoprotein (NP) alone is sufficient for this [12–15].

For a growing number of NNSVs, including rabies virus (RABV), vesicular stomatitis virus (VSV), and measles virus (MeV), IBs have been shown to have properties of liquid organelles [10, 11, 16], which are membraneless, liquid–liquid phase separated sub-compartments present in mammalian cells, such as the nucleolus or Cajal bodies in the cell nucleus, or stress granules in the cytoplasm (reviewed in [17, 18]). However, for EBOV IBs it is unclear whether they are indeed liquid organelles. In general, liquid organelle formation is driven by scaffold proteins forming a biomolecular condensate through interactions via intrinsically disordered regions (IDRs), multiple copies of interaction domains, or a combination of both (reviewed in [19]).

A number of properties define liquid organelles. One of these properties is that as membraneless structures liquid organelles can easily fuse with each other, and also rapidly react to external stimuli, resulting in their formation, restructuring, or dissolution [19, 20]. We have previously observed these behaviours for EBOV IBs, with smaller IBs fusing into larger ones during virus replication, and rapid changes being evident in form of massive dispersal followed by fusion during cell division [3]. Further, the surface area of IBs is minimized by forming a spherical shape, and due to the lack of limiting membranes, liquid organelles are in constant exchange with their surroundings, which can be demonstrated by fluorescence recovery after photobleaching (FRAP) of fluorescently labelled constituents of liquid organelles; however, these properties have so far not been demonstrated for EBOV IBs.

Here, we investigate the molecular mechanism underlying IB formation, and demonstrate in context of infectious virus that EBOV IBs fulfil all defining features of liquid organelles, and that their formation is mainly driven by oligomerization of NP, whereas both RNA binding and large parts of the IDRs in NP are not required for this process.

## Materials and methods

### Cells

VeroE6 and Huh7 cells (kindly provided by Stephan Becker, Philipps University-Marburg) as well as HEK 293T cells (Collection of Cell Lines in Veterinary Medicine CCLV-RIE1018) were maintained in Dulbecco’s Modified Eagle’s medium (DMEM) supplemented with 100 U/mL penicillin and 100 µg/mL streptomycin and 1x GlutaMAX (all Thermo Fisher Scientific) and fetal bovine serum (10% (DMEM10) for maintenance or 5% (DMEM5) for experiments) at 37°C with 5% CO_2_.

### Plasmids and viruses

Expression plasmids for VP30-GFP, VP35, flag-HA-VP35, NP, and NP-myc have been previously described [21, 22]. For cloning of constructs pCAGGS-NP_ΔIDR1-GS_, pCAGGS-NP_ΔIDR2.1-GS_, and pCAGGS-NP_ΔIDR2.2-GS_, two BamHI sites in the backbone of pCAGGS-NP were destroyed by PCR-amplifying the region between those two sites with the primers dBamHI-fwd: (CCAGCAGATCTGCATCTCAATTAGT) and dBamHI-rev (TGCAGCAGATCTAGACATGATAAGATAC), which mutate the BamHI sites into BglII sites (underlined), cutting the PCR-product with BglII, and ligating it into pCAGGS-NP cut with BamHI. Then, the relevant IDR-encoding regions were deleted and a linker encoding for GSGNLGS containing a BspEI site (encoding for the underlined SG dipeptide) and a BamHI site (encoding for the underlined GS dipeptide) was inserted via recombinant PCR. Finally, a synthetically produced DNA fragment encoding SGGGGS(G_4_S)_10_GGGGS was cloned into these constructs via BspEI and BamHI restriction sites. pCAGGS-NP_K160A.R171A.R174A_-myc, which is RNA binding-deficient, has been previously described [22]. pCAGGS-NP_Y21A.H22A_-myc, which is oligomerization-deficient, was generated by cutting NP with XhoI and BglII restriction enzymes and ligation with phosphorylated and hybridized oligonucleotides encoding the Y21A and H22A mutations. All generated constructs were verified by Sanger sequencing. Detailed cloning strategies are available upon request. The recombinant rgEBOV-VP30-GFP, which is based on Zaire ebolavirus rec/COD/1976/Mayinga-rgEBOV (GenBank accession number KF827427.1, rgEBOV) [23], and encodes a VP30 with GFP fused to its C-terminus instead of wildtype VP30, has been described before [21].

### Analysis of IB sphericity

Huh7 cells were seeded on 200 mesh Quantifoil^TM^ SiO2 R1.2/20 EM grids and infected with rgEBOV-VP30-GFP at an MOI of 0.1 in the BSL4 laboratory of the Friedrich-Loeffler-Institut (Greifswald-Insel Riems) following approved standard operating procedures. 14 hours post infection, cells were chemically fixed for 48 hours with 4% paraformaldehyde and 0.1% glutaraldehyde in PHEM buffer (25 mM HEPES, 10 mM EGTA, 2 mM MgCl2, 60 mM PIPES, pH 7.4), brought out of the BSL4 laboratory, and vitrified using a Leica EM GP2 automatic plunge-freezer. The infected cells were imaged using a Cryo-CLEM wide-field fluorescent microscope (Leica Microsystems). Z-stacks were acquired with a z-size of 30 μm and a z-step size of 300 nm resulting in 101 slices. To improve the resolution of the fluorescent signal, data deconvolution was performed with AutoQuant version X3.1.3 (Media Cybernetics) using a theoretical and adaptive point spread function for 100 iterations and the following optical parameters: lens immersion refractive index: 1; sample embedding refractive index: 1.31; sample distance from coverslip: 0 nm; emission wavelength of 525 nm (for GFP) and appropriate settings for the used objective (NA 0.9). Deconvolved fluorescence microscopy data was further analysed using Imaris software (Version 31 9.8.2, Oxford Instruments). Green fluorescent IBs of nine cells from three different grids (three cells per grid) were segmented in three dimensions using the surface segmentation algorithm of Imaris. The sphericity (ratio of the surface area of a sphere (with the same volume as the given IB) to the surface area of the IB) of each segmented IB (total number of segmented IBs: 344) was calculated using the Imaris statistics function.

### Sequence analysis

Prediction of amino acids in IDRs was done using the MEta-Server for protein Sequence Analysis (MESSA) [24]. Hydrophobicity according to Kyle/Doolittle [25] and charge at pH 7.0 was calculated as rolling average with a window size of 10 amino acids.

### FRAP analysis

For FRAP experiments VeroE6 or Huh7 cells were seeded in 4 – or 8-well chamber slides (IBIDI), and cells were either infected with rgEBOV-VP30-GFP at an MOI of 1 or transfected with 200 ng each of expression plasmids encoding for VP30-GFP, VP35, and NP or NP mutants. Medium was exchanged to DMEM5 1 hour post infection or 24 hours post transfection. Fluorescence recovery was analysed 16 hours post infection or 48 hours post transfection using a VisiScope Live Cell Imaging System with a 63x water immersion objective (Visitron Systems). Photobleaching was done using a 405 nm laser (20 mW), the FRAP time per pixel was set to 100 ms, and whole IBs were photobleached. Images were taken in 10 second intervals for 5 minutes, and image analysis was performed using the VisiView 4.3.0.1 (Visitron Systems) software.

### Westernblot analysis

NP and NP mutants were expressed in HEK 293T cells in 12-well format by transfecting 500 ng of the respective expression plasmid with TransIT LT-1 (Mirus Bio LLC) as recommended by the manufacturer, with 3 µl transfection reagent per µg DNA. One day after transfection, medium was exchanged to DMEM5 and samples were harvested on the following day. For this, cells were resuspended in 1 ml PBS, spun down for 5 min at 800 x g, and then boiled for 5 minutes at 95°C in 1x SDS-sample buffer. SDS-PAGE and semi-dry Western blotting was done as described before [26]. For analysis of expression either a polyclonal antibody against NP (rabbit, Gentaur, 0301-012) (for the IDR mutants) or a polyclonal antibody against c-myc (rabbit, Thermo Fisher, PA1-981) (for the point mutants) was used at a dilution of 1:5.000. As a loading control, a monoclonal antibody against Vinculin was used (mouse, Santa Cruz, sc-73614, 1:1.000). Secondary antibodies were goat anti-rabbit IgG IRDye 800CW (Li-cor, 926-32211) and goat anti-mouse IgG IRDye 680RD (Li-cor, 926-68070) at a dilution of 1:15.000. Blots were imaged with the Odyssey CLx system (Li-cor).

### Immunofluorescence analysis

Immunofluorescence analysis (IFA) was done as described before [27]. Briefly, Huh7 cells seeded on coverslips and transfected with 500 ng each of expression plasmids encoding for flag-HA-tagged-VP35, VP30-GFP and NP or NP mutants. 48 hours post transfection, cells were fixed, permeabilized, and stained with a monoclonal antibody against the flag-tag (mouse, Sigma-Aldrich 1:2000) and a polyclonal antibody against NP (rabbit, Gentaur, 0301-012, 1:100) (in case of the IDR mutants) or a polyclonal antibody against c-myc (rabbit, Thermo Fisher, PA1-981, 1:100) (in case of the RNA binding-deficient and the oligomerization-deficient mutant). Secondary antibodies were IgG goat anti-mouse Alexa Fluor568 (Thermo-Fisher, A-11031, 1:500) and anti-rabbit Alexa Fluor488 (Abcam, ab150077, 1:1.200). Nuclei were stained with DAPI using the ProLong Diamond Antifade Mountant (Invitrogen). Confocal image acquisition was done using a Leica SP5 with a 63x oil immersion objective (Leica Microsystems).

### Quantification

Confocal microscopy images were processed and analysed by a custom pipeline in the Vision4D 4.0 software (Arivis AG). Each fluorescence channel was denoised using the mean intensity filter. An intensity threshold segmenter was applied to detect individual cells with the percentile thresholding method set to 70% on the anti-myc (NP) channel. The IBs inside detected cells were identified using an adaptive mean intensity threshold segmenter with 50% local threshold and 35% split sensitivity. Fluorescence intensity and morphology of individual IBs in each channel was quantified. At least 5 images from each construct combination were quantified.

## Results

### EBOV IBs are liquid organelles

**Figure 1.**
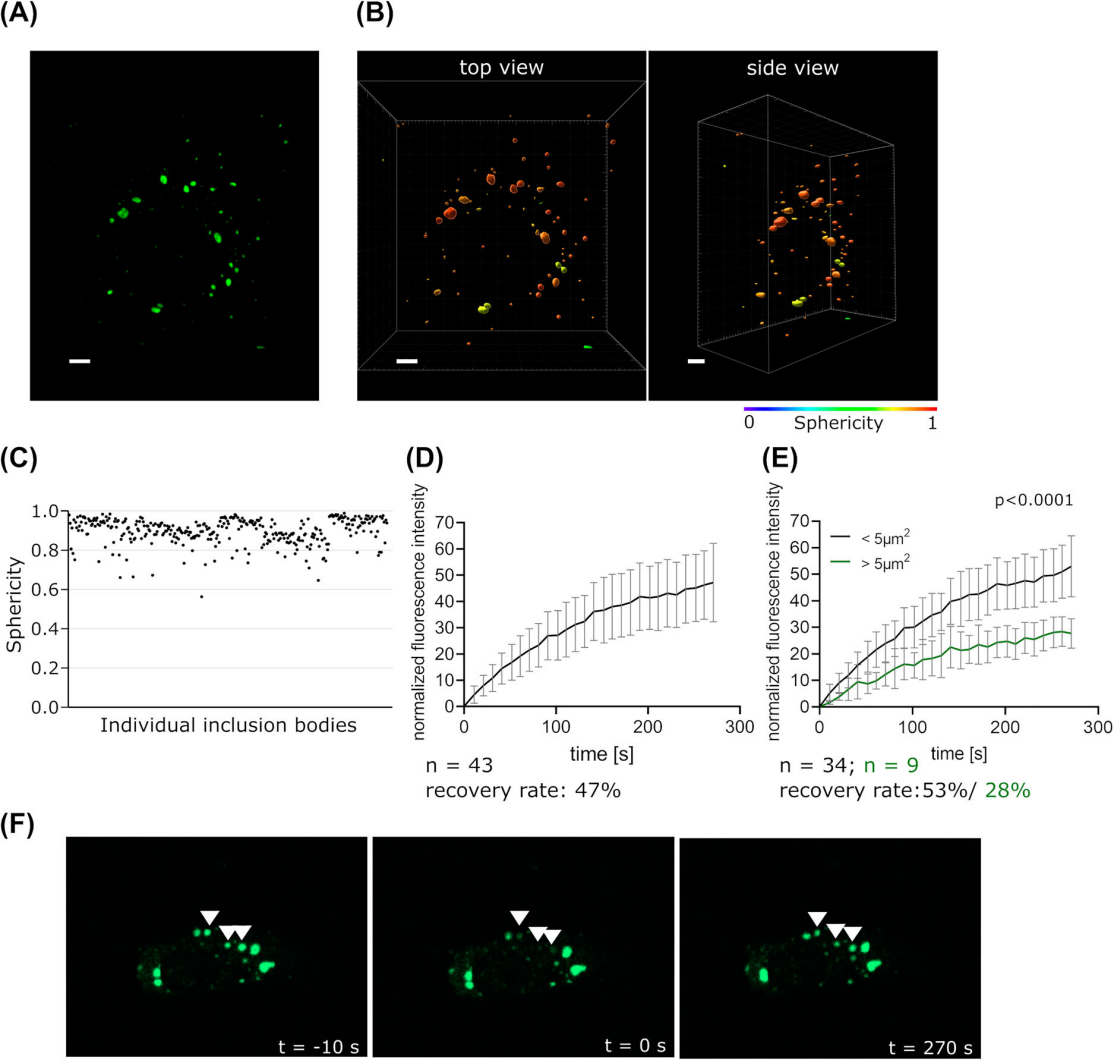
Characterization of liquid properties of EBOV IBs. (a) Representative fluorescence microscopy image of IBs (green) in an rgEBOV-VP30-GFP infected cell at 14 hours post infection. (b) Three-dimensional segmentation of the VP30-GFP signal of the same cell shown colour-coded according to sphericity. Scale bar = 5 µm. (c) Scatter plot of the sphericity of all segmented IBs (n = 344). (d) Fluorescence recovery of IBs. Cells were infected with rgEBOV-VP30-GFP at an MOI of 1, and at 16 hours post infection individual, whole IBs (marked with arrowheads) were photobleached and fluorescence recovery was monitored. (e) Quantification of fluorescence recovery of smaller (projected area < 5 µm^2^) and larger IBs (projected area > 5 µm^2^). p values of differences analysed by two-way ANOVA are shown. (f) Images of photobleached IBs 10 seconds before bleaching, immediately after bleaching (0 seconds), and 270 seconds after bleaching.

While we have previously demonstrated that EBOV IBs show some of the properties characteristic for liquid organelles, such as the ability to fuse and rapidly react to external stimuli [3], other properties such as spherical shape and constant exchange with surroundings still needed to be investigated to clarify whether EBOV IBs are indeed liquid organelles. Therefore, we analysed EBOV IBs in Huh7 cells infected with rgEBOV-VP30-GFP [28], which is a recombinant EBOV expressing a GFP-tagged form of the transcriptional activator VP30 that is recruited into IBs. Using this approach we observed that IBs exhibit a high degree of sphericity (Figure 1(a–c)).

To demonstrate a constant exchange of IB constituents with the surrounding cytoplasm, we performed FRAP experiments by photobleaching individual EBOV IBs that were labelled with VP30-GFP after infection of cells with rgEBOV-VP30-GFP, and observed a recovery of 47% fluorescence 270 seconds after photobleaching (Figure 1(d and f); Supplemental Video 1). As it has been shown for other NNSVs that IB size influences their liquid properties, with smaller IBs showing a more liquid behaviour and thus faster/higher fluorescence recovery, and larger IBs exhibiting a more solid behaviour with slower fluorescence recovery, we quantified fluorescence recovery of IBs with a projected area below and above 5 µm^2^ separately (Figure 1(e)). Indeed, also for EBOV IBs their size impacted their liquid properties, with a nearly 2-fold reduction in recovery for larger IBs compared to smaller ones. In summary, together with our previous observations [3] these results indicate that EBOV IBs are liquid organelles.

### IDRs are not a main contributing factor for IB formation

**Figure 2.**
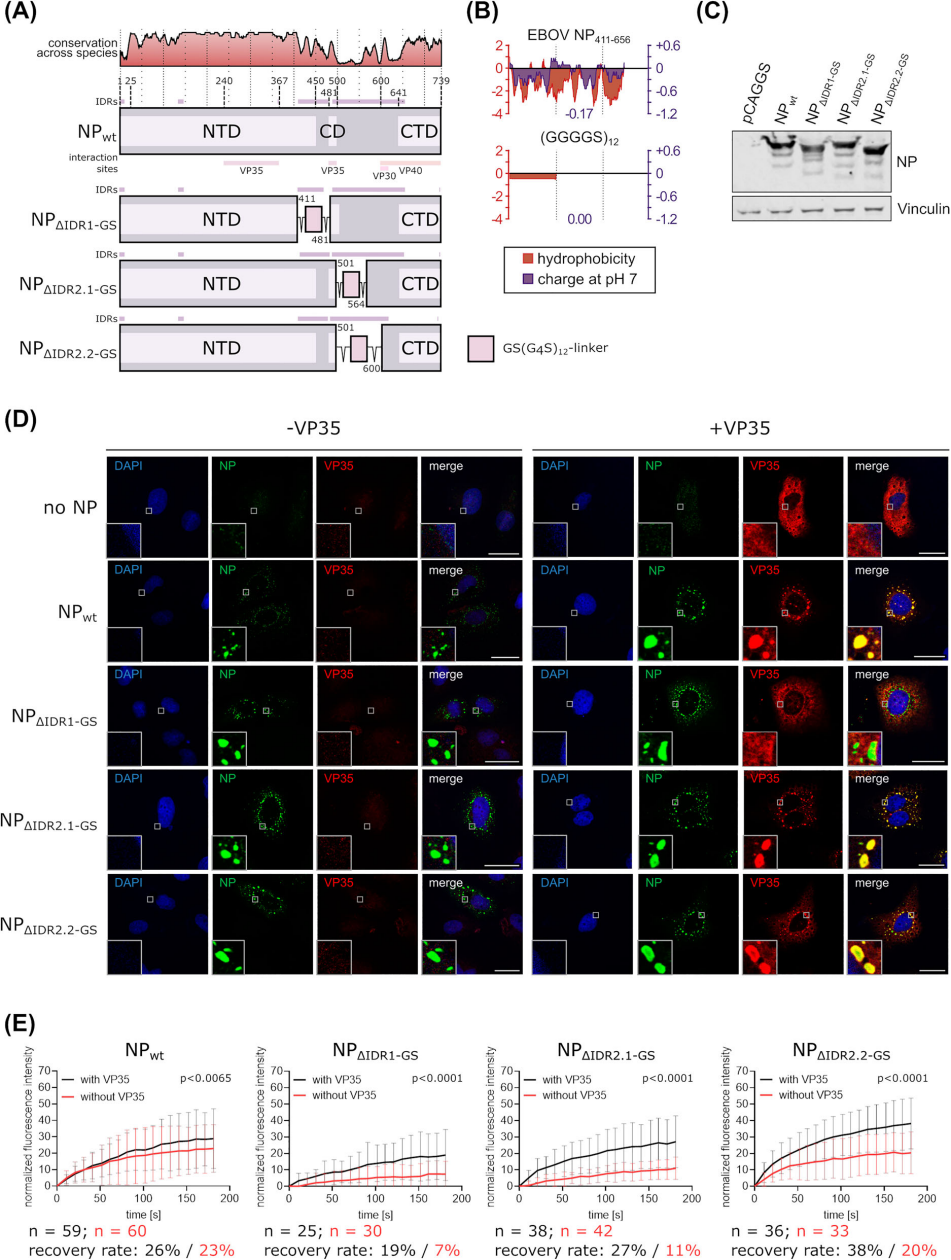
Characterization of the impact of IDRs on IB formation. (a) Schematic depiction of EBOV NP and NP mutants with amino acids in IDRs replaced by a flexible GS(G_4_S)_12_ linker. IDRs and known interaction sites with other viral proteins are shown. Amino acid conservation across ebolavirus species is indicated. NTD = N-terminal domain, CD = central domain, CTD = C-terminal domain (b) Hydrophobicity and charge at pH 7 of the region of EBOV NP containing the two large IDRs and of the GS(G_4_S)_12_ linker used to replace parts of this region. Hydrophobicity (according to Kyle/Doolittle) and charge was calculated as rolling average for a window size of 10 amino acids. (c) Expression of EBOV NP and NP mutants. Vinculin was used as a loading control. (d) Immunofluorescence imaging of NP or NP mutants. As a negative control the NP expression plasmid was omitted. Representative images out of two independent experiments with at least five images each per sample are shown. Scale bar = 30 µm. (e) Quantification of fluorescence recovery of IBs formed by NP or NP mutants. Cells were transfected with expression plasmids for NP or NP mutants, VP30-GFP, and either VP35 or empty vector, as indicated. p values of differences analysed by two-way ANOVA are shown.

IDRs are described as one of the driving determinants of liquid–liquid phase separation by engaging in multiple, often promiscuous, low-affinity interactions, including electrostatic, hydrophobic, cation-π, and π-π interactions (reviewed in [20, 29]). Since EBOV NP, which represents the main scaffold protein of EBOV IBs, can induce the formation of IBs on its own, we analyzed NP for the presence of IDRs. Sequence analyses using MESSA and comparing EBOV NP to several nucleo- and phosphoprotein of other NNSVs known to form IBs revealed that for EBOV the majority of IDRs is located in NP, whereas for other NNSV IDRs are predominantly found in the phosphoprotein (Supplemental Figure 1A). Specifically, EBOV NP has two major IDRs between amino acids A411 and L656 that surround its central domain (amino acids D481-N500) (Figure 2(a)). To test whether these IDRs are necessary for IB formation, we replaced them with a flexible GS(G_4_S)_12_ linker, which eliminates properties such as hydrophobicity and charge (Figure 2(b)) as well as the presence of aromatic amino acids in this region, i.e. properties responsible for the low affinity interactions of IDRs. For the first mutant the first, smaller IDR (amino acids A411-D481) was deleted and replaced by a GS(G_4_S)_12_ linker (NP_ΔIDR1-GS_). Deleting the second IDR was complicated by the fact that parts of it correspond to known interaction sites with viral proteins (i.e. the region from amino acid R600 onwards) [30], or were highly conserved across ebolavirus species, suggesting potentially important sequence motifs (i.e. the region between amino acids I565 and R600). We, therefore, generated 2 mutants, one deleting the second IDR from amino acid R501 only up to amino acid P564 (NP_ΔIDR2.1-GS_), and one deleting the IDR up to amino acid R600 (NP_ΔIDR2.2-GS_), and replacing it with a GS(G_4_S)_12_ linker. Comparable expression of these NP mutants was confirmed via Western blot (Figure 2(c)).

Next, IB formation by these NP mutants was tested in IFA. As VP35 has been described to enable the formation of IBs from NP mutants with C-terminal deletions that are unable to do so on their own [15], we performed these experiments in presence and absence of VP35. Surprisingly, all NP mutants still formed IBs, regardless of the presence or absence of VP35 (Figure 2(d)). However, in contrast to wildtype NP and the other mutants, the NP_ΔIDR1-GS_ mutant did not result in recruitment of VP35 into IBs, independent of the amount of VP35 (Figure 2(d), Supplemental Figure 1B, C), possibly because of deletion of amino acid D481, which is the first amino acid in the central domain known to interact with VP35 [15]. However, this mutant was still able to recruit VP30 into IBs (Supplemental Figure 1D), speaking against a gross misfolding of the protein.

Liquid properties of the IBs formed by NP mutants were tested by FRAP analysis in cells expressing these mutants together with VP30-GFP, and either VP35 or no VP35. A limited amount of fluorescence recovery was observed in all cases (Figure 2(e)). However, fluorescence recovery in IBs formed after transfection was lower than in IBs formed during infection, suggesting that in infection additional viral or host proteins support a more liquid character of IBs, and, indeed, for all mutants addition of VP35 resulted in an increase in fluorescence recovery (Figure 2(e)).

### RNA binding is not required for IB formation

**Figure 3.**
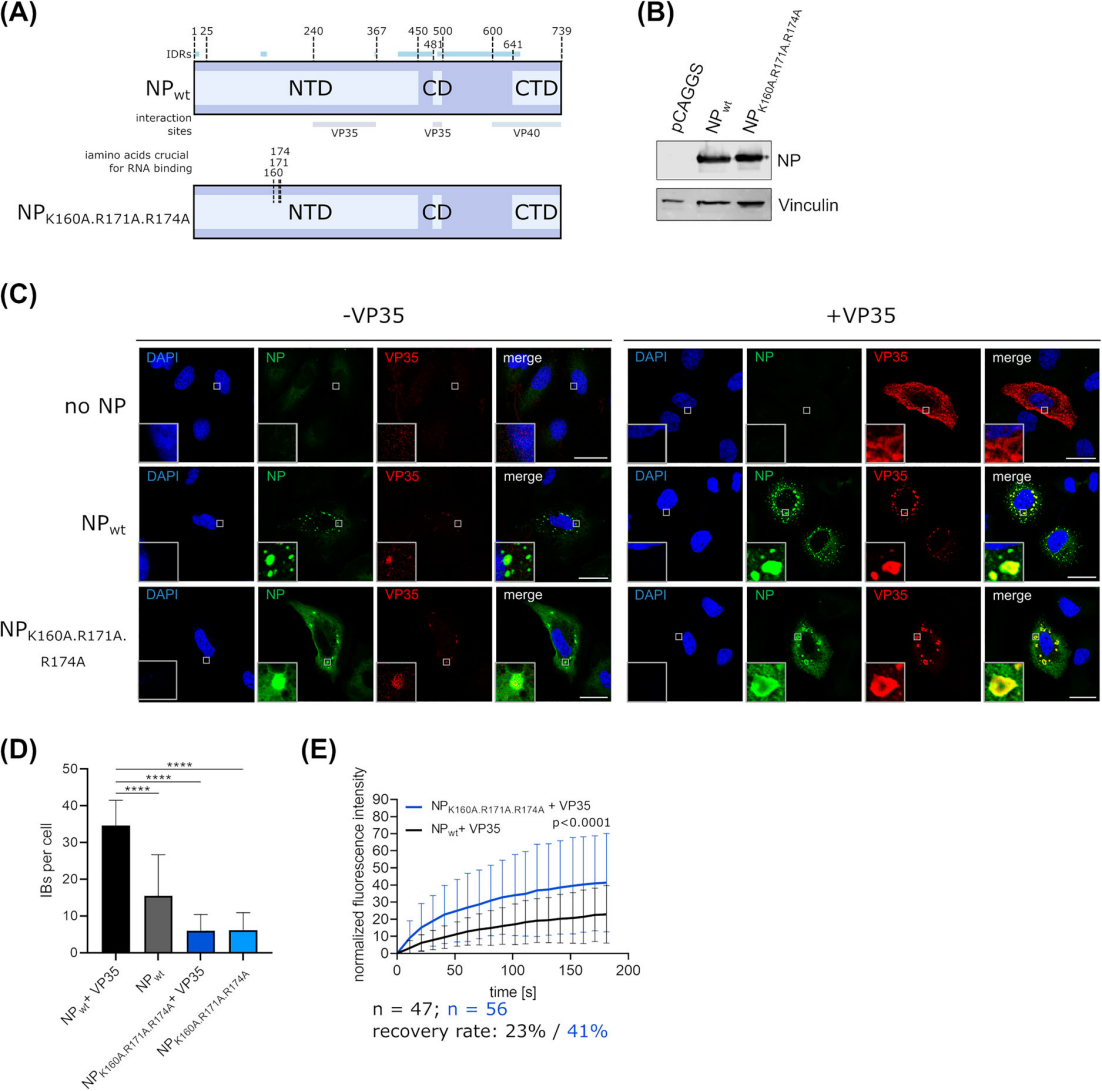
Role of RNA binding for IB formation. (a) Schematic depiction of EBOV NP and NP mutants with point mutations in amino acids necessary for RNA binding. IDRs and known interaction sites with other viral proteins are shown. (b) Expression of EBOV NP and an RNA binding-deficient NP mutant. Vinculin was used as a loading control. (c) Immunofluorescence imaging of wildtype NP or an RNA binding-deficient NP mutant expressed in cells in presence or absence of VP35. As a negative control the NP expression plasmid was omitted. Representative images out of two independent experiments with at least five images each per sample are shown. Scale bar = 30 µm. (d) Quantification of IBs per cell. IBs formed by expression of EBOV NP or an RNA binding-deficient NP in presence and absence of VP35 were counted. (e) Quantification of fluorescence recovery of IBs formed by wildtype NP or an RNA binding-deficient NP mutant. Cells were transfected with expression plasmids for VP35, VP30-GFP, and NP or the NP mutant as indicated. p values of differences analysed by two-way ANOVA are shown.

One of the main functions of NP is to bind viral genomic RNA, and NP is known to also bind cellular RNAs, particularly when no viral RNAs are available. Therefore, we used an NP mutant that we and others have previously shown to no longer bind to RNA [22, 31, 32] to investigate whether RNA-binding of NP influences IB formation (Figure 3(a)). Expression of this mutant was comparable to NP_wt_ (Figure 3(b)), and IFA showed that it was still able to form IBs, and to recruit VP35 into IBs (Figure 3(c)). However, the number of IBs per cell was reduced (Figure 3(d)), and in contrast to wildtype NP, which was almost exclusively found in IBs, the RNA binding-deficient NP mutant showed, besides clear accumulation in IBs, also a diffuse distribution throughout the cytoplasm, albeit at a low level (Figure 3(c)). Interestingly, FRAP analysis of these IBs showed that RNA binding-deficient NP results in a higher fluorescence recovery than wildtype NP (Figure 3(e)).

### Oligomerization of NP is necessary for IB formation

**Figure 4.**
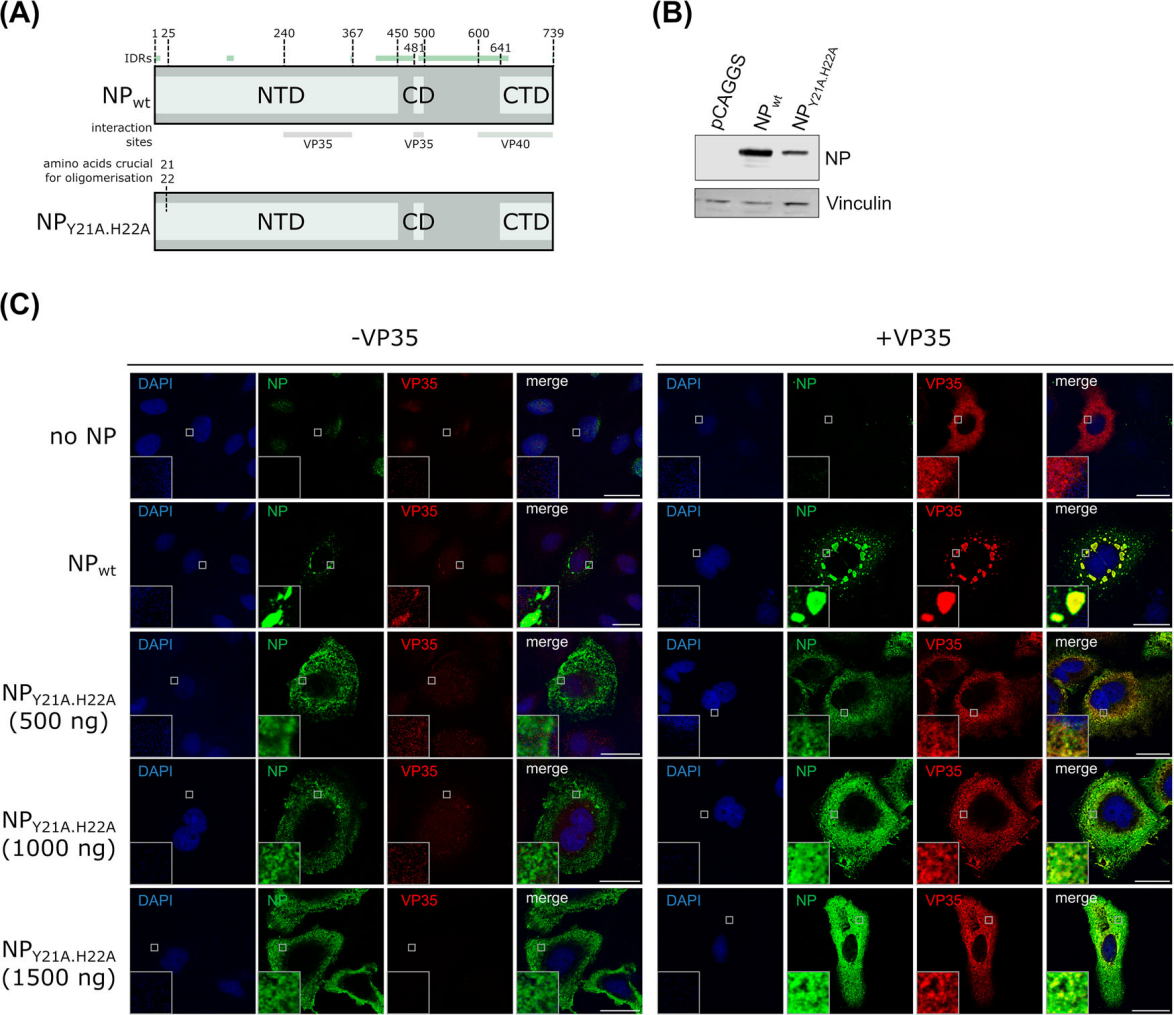
Role of oligomerization for IB formation. (a) Schematic depiction of EBOV NP and NP mutants with point mutations in amino acids necessary for oligomerization. IDRs and known interaction sites with other viral proteins are shown. (b) Expression of EBOV NP and an oligomerization-deficient NP mutant. Vinculin was used as a loading control. (c) Immunofluorescence imaging of NP or an oligomerization-deficient NP mutant (transfected amounts as indicated) expressed in cells in presence or absence of VP35. As a negative control the NP expression plasmid was omitted. Representative images out of three independent experiments with at least two images each per sample are shown. Scale bar = 30 µm.

Besides IDRs also multiple copies of interacting domains can contribute to biomolecular condensation of scaffold proteins, although NP does not contain identical copies of the same interactions site. We, therefore, investigated to what extend oligomerization of NP contributes to IB formation using NP_Y21A.H22A_, a known oligomerization-deficient mutant of NP [32] (Figure 4(a)). While readily expressed, albeit at slightly lower levels than wildtype NP (Figure 4(b)), this mutant was not able to form IBs, even in presence of VP35, and also when increasing the amount of transfected NP plasmid (Figure 4(c)).

## Discussion

Liquid–liquid phase separation is increasingly recognized as a mechanism by which NNSVs partition their IBs from the cytoplasm [10, 11, 16]. The data presented in this study together with our previous work [3] show that EBOV IBs exhibit the defining properties of liquid organelles: 1) they are spherical structures, that 2) can fuse with each other as previously shown, 3) are in constant exchange with their surroundings as demonstrated by fluorescence recovery, and 4) can rapidly react to external stimuli as seen by the previously observed extensive restructuring of IBs during mitosis.

Interestingly, for the formation of EBOV IBs the presence of NP as main scaffold protein is sufficient [12, 13, 30], in contrast to other NNSVs, where both the nucleoprotein and the phosphoprotein are required [5, 10, 11]. A potential explanation is that in EBOV NP more regions are intrinsically disordered than in other NNSV nucleoproteins, whereas for those viruses it is rather the phosphoprotein that contains the majority of amino acids contributing to IDRs. Potentially supporting this idea, Miyake et al. could show that deleting C-terminal parts of NP (including parts of the second IDR) abolished IB formation, but that this phenotype could be reverted by addition of VP35, raising the possibility that the IDRs in VP35 compensate for the deleted parts of the NP IDR [15]. However, in these experiments the C-terminal domain of NP was always deleted, making it impossible to distinguish an effect of this domain vs. an effect of the IDR. Our data now show that replacement of the NP IDRs by a linker (so that the CTD is still present in these NP mutants) does not inhibit IB formation (Figure 2(d) and Supplemental Figure 1B), indicating that IDRs are not the main contributing factor in IB formation. These data can be reconciled with the findings of Miyake et al. by postulating that the CTD of NP plays a role in IB formation, which can be substituted for by VP35, although the mechanism for this remains unclear. Another possible explanation for the difference in requirements for IB formation between EBOV and other NNSVs is that for EBOV it is oligomerization of NP that drives this process, as mutating only two amino acids described to be necessary for oligomerization completely abolished formation of IBs in a way that also could not be rescued by VP35. In contrast to this, for RABV it could be shown that a large IDR in P is essential for IB formation [10], suggesting intriguing differences in how different NNSVs accomplish phase separation of IBs.

However, IDRs do influence the behaviour of IBs, as apparent by changes in fluorescence recovery for mutants where IDRs had been replaced. Similarly, other viral proteins such as VP35 influence the liquid behaviour of IBs and result in higher fluorescence recovery, suggesting that VP35 “liquifies” these structures. Given that VP35 has been shown to chaperone monomeric NP and prevent oligomerization [33], it is possible that the presence of VP35 shifts the balance of oligomeric and monomeric NP in IBs, and in doing so facilitates this “liquefaction”. Interestingly, this is also the case for the mutant NP_ΔIDR1-GS_, which is limited in its interaction with VP35, as we no longer see accumulation of VP35 in IBs, but rather a distribution throughout the cytoplasm (including the regions occupied by IBs). However, while in this mutant the interaction between the C-terminal portion of VP35 and the CD of NP is affected by the deletion of amino acid D481 [15], the interaction between the N-terminal part of VP35 and the NTD of NP, which is crucial for preventing NP from oligomerizing [33], is unaffected, possibly explaining why this mutant still has “liquefying” properties.

Besides VP35 also other viral factors influence the behaviour of IBs, as IBs in infected cells show significantly higher fluorescence recovery than those in cells after transfection of only NP, VP30-GFP and VP35. While the exact nature of these factors is unknown, the fact that RNA-binding seems to “solidify” IBs (as RNA binding-deficient versions of NP result in IBs with higher fluorescence recovery) suggests that rather than RNA it is viral proteins (either directly or through recruitment of additional host cell factors and/or posttranslational modifications) that are responsible for this phenotype.

Further, the fact that RNA binding seems to solidify IBs implies that viral RNA synthesis should result in such a solidification. Indeed, the fact that larger, and thus presumably “older” IBs show less fluorescence recovery supports this idea. Interestingly, this seems to be a more common phenomenon among NNSV, as also for MeV IBs there is a negative correlation between IB size and fluorescence recovery [11].

Besides viral factors also a number of host factors have been shown to be associated with IBs, and in some cases their function has been characterized. For example, the host nuclear RNA export factor 1 (NXF1) has been shown to export viral mRNA out of inclusion bodies [22, 26], and it is tempting to speculate that the need for this host factor might be due to the fact that the phase separation of IBs generates an environment similar to nuclear phase-separated environments, including the nuclear pore. However, so far no IB-associated host factors are known to contribute directly to IB formation or their liquid properties. Similarly, a possible role of the cytoskeleton in EBOV IB formation should be addressed in future studies, as for RABV inhibition of actin polymerization has been shown to impact IB formation, and both EBOV and RABV nucleocapsids can associate with actin filaments [10, 34]. Finally, another non-viral factor that could influence the liquid properties of IBs is temperature, and while all experiments in this study were done at 37°C, different temperatures (as they might occur during disease or in the natural host) could certainly influence the behaviour of EBOV IBs.

Overall, while our study has established EBOV IBs as liquid organelles and given a first insight into the mechanisms underlying their formation, further studies are required to better understand these mechanisms and the viral and possibly host factors that contribute to it, and also the progression of IBs during the course of infection, e.g. possible changes in characteristics, constituents, and maybe even function, will be an important topic for future investigation.

## Supplementary Material

Supplemental Material

Supplemental Material

## Data Availability

All data supporting the findings of this study are available within the article and its supplementary material.
