# Supplementary material for: Ebola virus inclusion bodies are liquid organelles whose formation is facilitated by nucleoprotein oligomerization
Source: Emerg Microbes Infect. 2023 Jun 22;12(2):2223727. doi: 10.1080/22221751.2023.2223727 (PMC10288931; doi:10.1080/22221751.2023.2223727)
Supplement: Supplemental Material [file TEMI_A_2223727_SM1816.pdf]

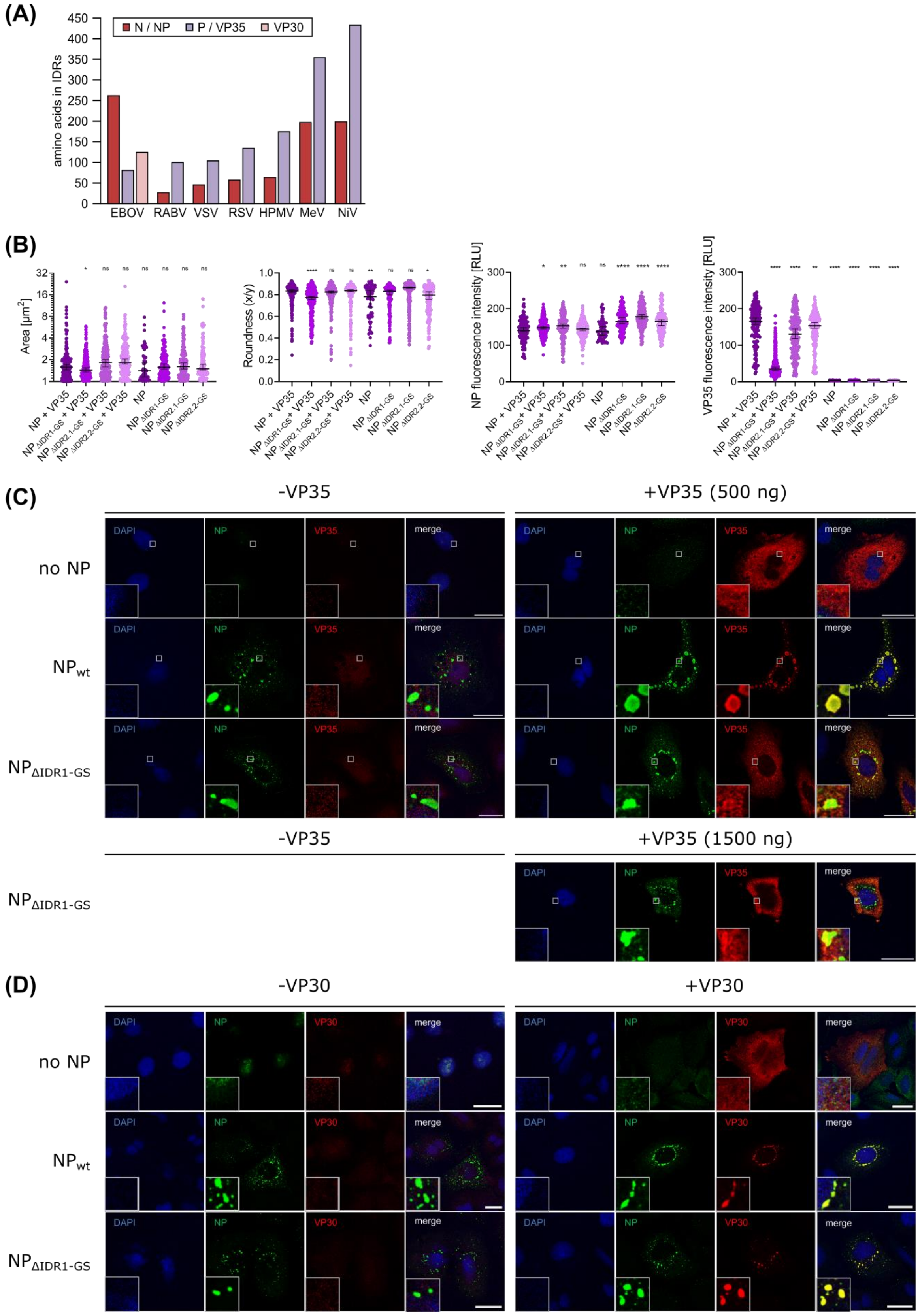

Supplemental Figure 1. Supplementary data to Figure 2. (A) Amino acids in IDRs in nucleo- and phosphoproteins of various NNSV. IDRs were predicted using the MEta Server for Sequence Analysis (MESSA). (B) Quantitative analysis of inclusion bodies formed by NP and

NP mutants with amino acids replaced by a flexible $GS(G_4S)_{12}$ linker in presence or absence of VP35. Two independent experiments with at least five images per sample were analysed, and IB size, roundness and NP/VP35 fluorescence intensity was quantified. (C) Interaction of $NP_{\Delta IDR1\text{-}GS}$ with VP35. Immunofluorescence imaging of wildtype NP or the NP mutant $NP_{\Delta IDR1\text{-}GS}$ expressed in cells with or without VP35 in amounts as indicated. As a negative control the NP expression plasmid was omitted. Representative images from three independent experiments with at least two images per sample are shown. Scale bar = 30 µm. (D) Interaction of $NP_{\Delta IDR1\text{-}GS}$ with VP30. Immunofluorescence imaging of wildtype NP or the NP mutant $NP_{\Delta IDR1\text{-}GS}$ expressed in cells with or without VP30 as indicated. As a negative control the NP expression plasmid was omitted. Representative images from two independent experiments with at least five images per sample are shown. Scale bar = 30 µm.

Supplemental Video 1. Fluorescence recovery after photobleaching of inclusion bodies (IBs) formed in rgEBOV-VP30-GFP infected VeroE6 cells.
