# Supplementary material for: Ebola virus inclusion bodies are liquid organelles whose formation is facilitated by nucleoprotein oligomerization
Source: Emerg Microbes Infect. 2023 Jun 22;12(2):2223727. doi: 10.1080/22221751.2023.2223727 (PMC10288931; doi:10.1080/22221751.2023.2223727)
Supplement: Supplemental Material [file TEMI_A_2223727_SM1815.pptx]

## Slide 1
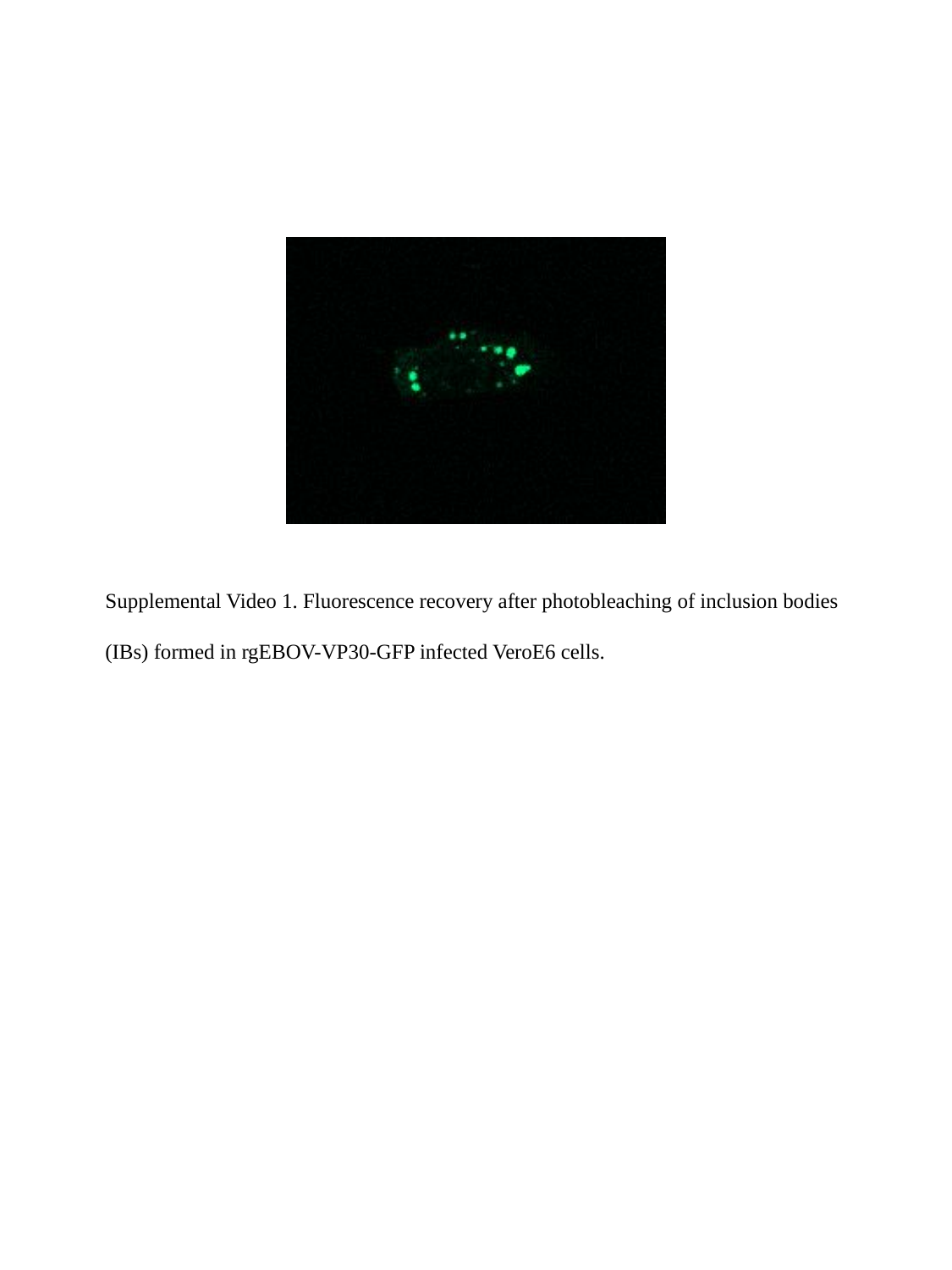

Supplemental Video 1. Fluorescence recovery after photobleaching of inclusion bodies (IBs) formed in rgEBOV-VP30-GFP infected VeroE6 cells.
